# Assessing the performance of quantum-mechanical descriptors in physicochemical and biological property prediction

**DOI:** 10.1039/d5dd00411j

**Published:** 2026-01-19

**Authors:** Alejandra Hinostroza Caldas, Artem Kokorin, Alexandre Tkatchenko, Leonardo Medrano Sandonas

**Affiliations:** a Universidad Nacional de Ingeniería Av. Túpac Amaru 210, Rímac Lima 15333 Peru; b Department of Physics and Materials Science, University of Luxembourg L-1511 Luxembourg City Luxembourg alexandre.tkatchenko@uni.lu leonardo.medrano@tu-dresden.de

## Abstract

Machine learning (ML) approaches have drastically advanced the exploration of structure–property and property–property relationships in computer-aided drug discovery. A central challenge in this field is the identification of molecular descriptors that can effectively capture both geometric- and electronic structure-derived features, enabling the development of reliable and interpretable predictive models. While numerous descriptors focusing solely on structural characteristics have been recently proposed, improvements in model accuracy often come at the cost of increased computational demands, thereby restricting their practical applicability. To address this challenge, we introduce the “QUantum Electronic Descriptor” (QUED) framework, which integrates both structural and electronic data of molecules to develop ML regression models for property prediction. In doing so, a quantum-mechanical (QM) descriptor is derived from molecular and atomic properties computed using the semi-empirical density functional tight-binding (DFTB) method, which allows for efficient modelling of both small and large drug-like molecules. This descriptor is combined with inexpensive geometric descriptors—capturing two-body and three-body interatomic interactions—to form comprehensive molecular representations used to train Kernel Ridge Regression and XGBoost models. As a proof of concept, we validate QUED using the QM7-X dataset, which comprises equilibrium and non-equilibrium conformations of small drug-like molecules, demonstrating that incorporating electronic structure data notably enhances the accuracy of ML models for predicting physicochemical properties. For biological endpoints, we find that QM properties provide some predictive value for toxicity and lipophilicity prediction, as assessed using the TDCommons-LD_50_ and the MoleculeNet benchmark datasets. Moreover, a SHapley Additive exPlanations (SHAP) analysis of the toxicity and lipophilicity predictive models reveals that molecular orbital energies and DFTB energy components are among the most influential electronic features. Hence, our work underscores the importance of incorporating QM descriptors to enhance both the accuracy and interpretability of ML models for predicting multiple properties relevant to pharmaceutical and biological applications.

## Abstract 2

QUED, a QM/ML framework that combines structural and electronic molecular information to build regression models for physicochemical and biological property prediction. Our work highlights the value of QM data for reliable and interpretable models.

## Graphical abstract

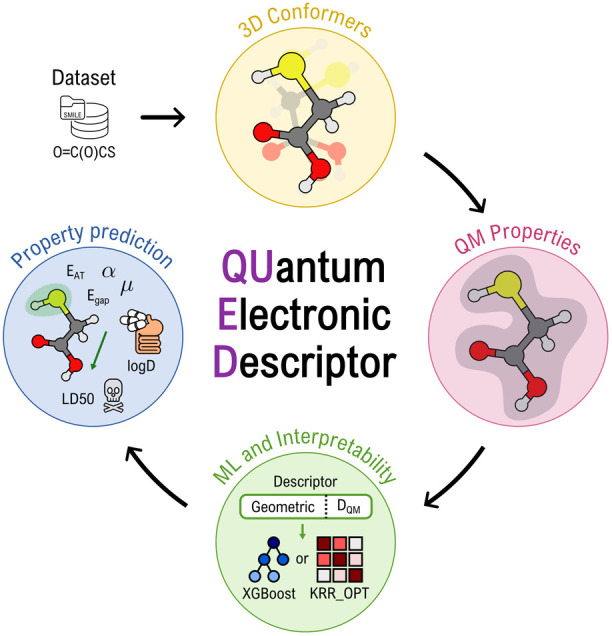

## 1 Introduction

Quantum-mechanical (QM) descriptors have emerged as powerful tools in molecular property prediction, effectively bridging theoretical physics and chemistry with practical applications in drug discovery^1–4^ and material science.^5–8^ These descriptors are derived from the electronic structure of molecules, which is obtained by solving the Schrödinger equation. While this process can be computationally demanding—particularly for large and flexible drug-like molecules—advancements in computational chemistry have made it more tractable through approximate methods such as density functional theory (DFT)^9,10^ and semi-empirical (SE) methods.^11,12^ A critical input for these QM methods is the 3D spatial arrangement of atoms forming a molecular system, which is often overlooked in the development of predictive models within computer-aided drug discovery.^13,14^ Traditional descriptors used in this context are typically based on easily computable molecular and atomic features, such as SMILES (Simplified Molecular Input Line Entry System) strings,^15^ molecular weight,^16^ Morgan fingerprints,^17^ and Fukui functions.^18^ Despite their practicality, these descriptors lack the mechanistic insight into short- and long-range molecular interactions that electronic structure-based descriptors can provide. As a result, QM descriptors are attracting increasing attention for predicting physicochemical properties,^8,19^ environmental-related properties,^20,21^ and ADMET (Absorption, Distribution, Metabolism, Excretion, and Toxicity) endpoints.^22–25^

Among the ADMET endpoints, toxicity stands out as a critical factor due to its direct implications for drug safety, spanning from mild adverse effects to severe, life-threatening outcomes. Indeed, pre-clinical and clinical (animal and human) toxicity issues account for over 30% of drug attrition,^26^ highlighting the urgent need for reliable early-stage prediction methods. Late-stage failures not only entail significant losses in time and resources but also often require revisiting earlier development phases, even when compounds have shown favorable pharmacokinetic properties.^27,28^ Consequently, computer-aided screening is essential for identifying potential toxicity risk early in the drug discovery pipeline, thereby supporting more efficient compound prioritization and structural optimization. Recent studies have emphasized the predictive value of descriptors that encode electronic properties, such as molecular orbital energies, polarization, reactivity, and total energy, in modelling toxicity across various biological datasets.^29–34^ These features are particularly relevant for capturing complex covalent interactions, which are central to many toxicological endpoints. Moreover, the increasing availability of large and curated toxicity datasets (*e.g.*, ToxBenchmark,^35^ MoleculeNet^36^) has spurred the development of numerous machine learning (ML) frameworks for toxicity prediction.^15,37,38^

A critical challenge in developing ML models for property prediction that incorporate conformational sampling lies in the structural representation of conformers. Specifically, one must define a multidimensional function that transforms discrete atomic information—such as Cartesian coordinates and nuclear charges—into a task-appropriate structural representation, commonly referred to as a geometric descriptor^39,40^ These transformations must satisfy several essential criteria: they should preserve fundamental physical symmetries (*i.e.*, be invariant under translations, rotations, and permutations of identical atoms), ensure smoothness (so that small changes in atomic positions lead to small changes in the descriptor), and be complete (ensuring that distinct molecular configurations are not mapped to the same representation).^41,42^ Early examples of such descriptors include the Coulomb Matrix (CM)^43^ and the Bag-of-Bonds (BOB),^44^ which achieve rotational and translational invariance by encoding two-body coulombic interactions. Building on these foundations, more expressive and permutationally invariant many-body descriptors^45^—such as the Spectrum of London and Axilrod–Teller–Muto (SLATM) potentials^46^ and the Faber–Christensen–Huang–Lilienfeld (FCHL) representation—have demonstrated higher accuracy in predicting both extensive and intensive physicochemical properties of small drug-like molecules. More sophisticated descriptors have since been introduced,^39,47^ offering even greater predictive performance. However, these improvements often come at the expense of increased computational cost, particularly when applied to large and flexible molecules common in pharmaceutical and biological contexts. To address this challenge in the development of ML models for biomedical endpoint prediction, one promising strategy is to augment inexpensive geometric descriptors with QM-derived features. This hybrid approach can enhance both accuracy and generalizability while maintaining computational efficiency. Furthermore, due to their inherent simplicity and informative nature, such combined descriptors may also facilitate the development of more interpretable ML models for toxicity prediction.^48^

**Fig. 1:**
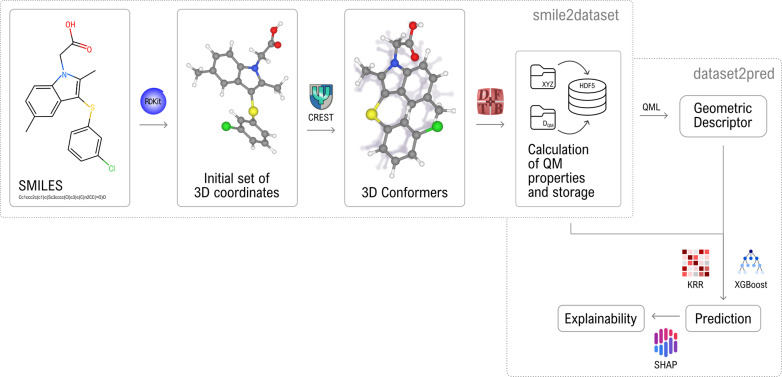
Diagram of the “Quantum Electronic Descriptor” (QUED) framework. The input to QUED consists of a set of molecules represented as SMILES strings. Three-dimensional molecular structures are then generated using the RDKit package. Conformational ensembles and quantum-mechanical (QM) properties for each molecule are subsequently computed using the CREST and DFTB+ codes. This resulting dataset forms the basis for constructing geometric descriptors, which are then used to predict molecular properties or to train machine learning (ML) models *via* regression methods such as Kernel Ridge Regression (KRR) and eXtreme Gradient Boosting (XGBoost). The choice of descriptor depends on the specific regression task.

In this work, we introduce the “QUantum Electronic Descriptor” (QUED) framework, which integrates structural features and electronic structure data of molecules to develop ML regression models for property prediction (see Fig. 1). Our primary goal is to evaluate the performance and interpretability of these hybrid descriptors in predicting physicochemical properties and biological responses of drug-like molecules. To this end, we employ inexpensive structural descriptors such as BOB and SLATM to capture essential geometrical information. These are complemented by an electronic descriptor (*D*_QM_), composed of molecular and atomic properties computed using the semi-empirical QM method density functional tight-binding (DFTB).^49^ Both types of descriptors are used in combination with kernel ridge regression (KRR) and XGBoost algorithms to investigate different strategies for enhancing predictive reliability. As a proof-of-concept, we first assess QUED ability to predict extensive and intensive physicochemical properties of both equilibrium and non-equilibrium small molecules from the QM7-X dataset.^50^ The gained insights are then leveraged to evaluate the effectiveness of hybrid descriptors for toxicity prediction in large and flexible drug-like molecules from the LD_50_ dataset.^51,52^ We further explore QUED potential to predict lipophilicity using data from the MoleculeNet benchmark.^36^ To interpret model predictions and identify key electronic features relevant to target properties, we employ SHapley Additive exPlanations (SHAP).^53^ Our results demonstrate that DFTB-derived electronic descriptors capture subtle molecular interactions that purely geometrical representations often miss, thereby enhancing the performance of specific ML regression models. In particular, molecular orbital energies and DFTB energy components are key contributors to this improvement. Overall, QUED offers a robust and interpretable framework for developing predictive models of physicochemical properties and biological responses.

## 2 Methods

### 2.1 The QUED framework

#### 2.1.1 Machine learning regression techniques

##### 2.1.1.1 Kernel ridge regression (KRR)

As part of the QUED framework, we have developed the 
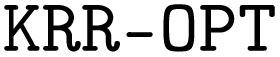
 toolbox that can be used to train ML models for property prediction using the KRR method (also known as the ‘kernel trick’).^54^ Various features, including kernel functions, molecular descriptors, and metrics, have been implemented to capture the unknown structure-to-property relationships in complex molecular systems. 
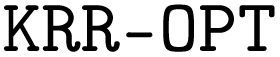
 toolbox also considers a quasi-Newton algorithm such as the limited-memory Broyden–Fletcher–Goldfarb–Shanno (BFGS) for hyperparameter optimization. Within 
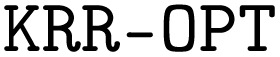
, the target property array ŷ is given as1
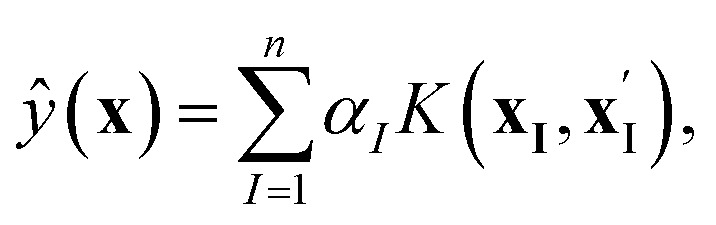
where **x** and **x′** denote the chosen representation of the molecules, *K* is the kernel function, and *α* = (**K** + *λ***I**)^−1^**y** is the solution to the minimization problem,2
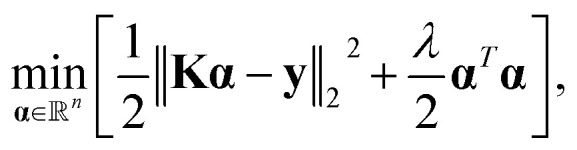
with *λ* as a small and mathematically necessary regularization parameter, which secures the invertibility of the kernel matrix. In this work, we have only considered the Laplacian and Gaussian kernel functions, which are represented as,3
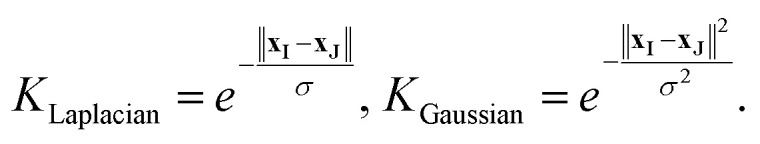
*σ* is the length-scale hyperparameter, and the second hyperparameter to be optimized in the 
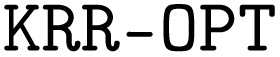
 algorithm. The determination of the optimal set of hyperparameters relies on the simultaneous optimization of the hyperparameters, training set, and validation set. To carry out this process, 
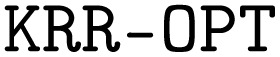
 considers a given number of randomly distributed molecular sets. To train the KRR models, each benchmark dataset was randomly split into training, validation, and test sets. The number of samples allocated to the training and validation sets is reported in Table S2 of the SI, while the remaining samples were used for testing. The 
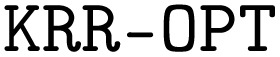
 toolbox can be accessed in the QUED Github repository.

##### 2.1.1.2 eXtreme gradient boosting (XGBoost)

As a member of the gradient boosting category, at each boosting iteration, the XGBoost algorithm^55^ augments the model with a new tree *f*_*t*_(*x*) by minimizing a regularized objective function given by4
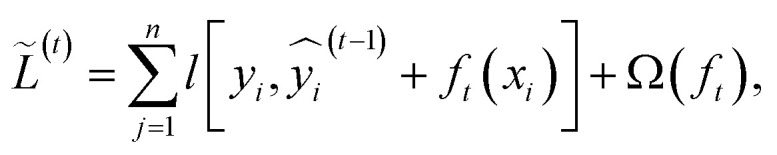
where *l* represents the loss function measuring discrepancy between the true label *y*_*i*_ and the prediction ŷ^(*t*−1)^_*i*_, while Ω(*f*_*t*_) penalizes model complexity. To simplify eqn (4)'s optimization problem, the loss is approximated by a second-order Taylor expansion,5
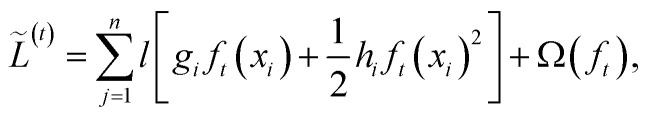
where *g*_*i*_ and *h*_*i*_ denote the first and second derivatives (gradient and Hessian) of the loss with respect to the prediction for the *i*th sample. The contributions from samples falling into each leaf node *j* are then aggregated, leading to6
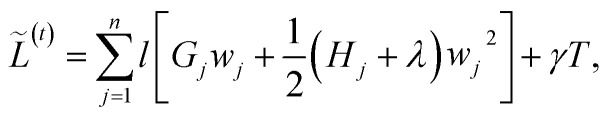
with 
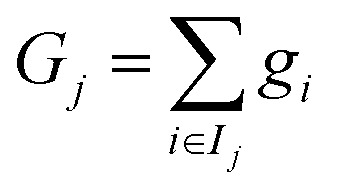
 and 
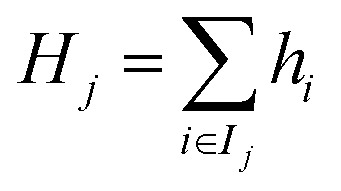
 being the sum of gradients and Hessians of all samples in leaf *j*, *λ* and *γ* acting as regularization parameters, and *T* denoting the total number of leaves. Optimizing eqn (6) with respect to the leaf weight *w*_*j*_ by setting its derivative to zero yields the optimal weight7
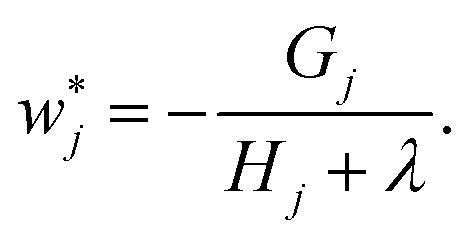


Hyperparameter tuning was executed *via* a Bayesian optimization framework implemented in the Optuna^56^ package. In each iteration, a five-fold cross-validation was employed, with the objective of maximizing the negative root mean square error. Furthermore, each predictive model underwent 100 iterations of this optimization process. For all benchmark datasets, the training set sizes matched those used to develop the KRR models. Training samples were here selected using the farthest point sampling (FPS) technique,^57^ and the remaining samples constituted the test set. More details about the hyperparameter optimization can be found in Table S3 of SI. The module performing XGBoost calculations will be integrated into the 
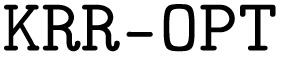
 toolbox in a later version.

#### 2.1.2 Molecular descriptors

**Fig. 2:**
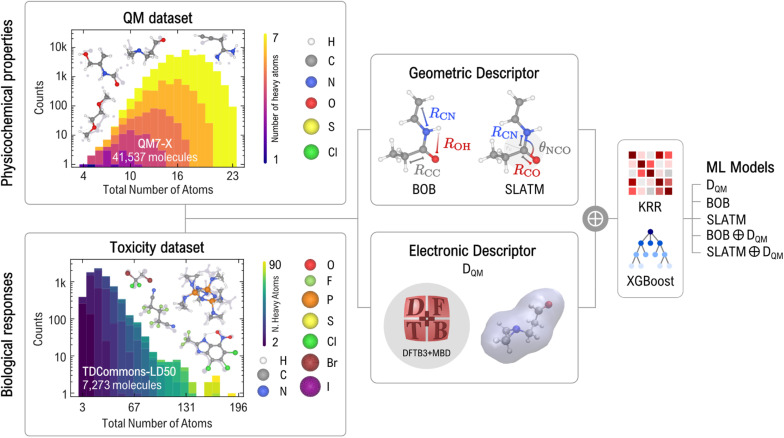
Scheme of the QUED framework used to predict physicochemical and biological properties from structural and electronic descriptors. QUED integrates geometric representations, such as Bag-of-Bonds (BOB) and the Spectrum of London and Axilrod–Teller–Muto potential (SLATM), with a quantum mechanical (QM) descriptor (*D*_QM_) computed at the DFTB3 + MBD level. Predictive models are trained using Kernel Ridge Regression (KRR) and eXtreme Gradient Boosting (XGBoost) methods on two datasets, including QM7-X^50^ for physicochemical properties of small organic molecules and TDCommons-LD_50_ (ref. 51) for experimental acute toxicity values of drug-like compounds. Molecular size distributions and elemental compositions are shown for these datasets. We have also considered the MoleculeNet-Lipophilicity benchmark dataset,^36^ plots are provided in Fig. S6 of SI.

A crucial step in developing ML regression models within the QUED framework includes the measurement of molecular similarity through the comparison of high-dimensional molecular descriptors. We have here evaluated the predictive performance of geometric and electronic descriptors both independently and in combination (*via* concatenation) to predict physicochemical properties and biological responses of drug-like molecules. An overview of the molecular descriptors used in this work, and their integration into QUED, is illustrated in Fig. 2. Details of the computational costs involved in generating the electronic and geometric descriptors are provided in Fig. S5 of the SI.

##### 2.1.2.1 Geometric representations

The two-body molecular descriptor Bag-of-Bonds (BOB), as well as the two and three-body descriptor SLATM, were used in the present work. BOB, a vectorized molecular representation, was introduced as a slightly more complex and improved version of the Coulomb Matrix (CM) representation,^43^ inspired by an ML approach in text processing bag-of-words.^44^ This descriptor is obtained by sorting into ‘bags’ (*i.e.*, individual vectors) the types of bonds between pairs of atoms, which comprise a CM element. Each bag contains a single type of bond, and the bags are concatenated; the end of the vector is padded with zeros, so as to obtain the same vector length independent of the size of the molecules in a given dataset. However, the BOB representation is solely based on the atomic numbers and interatomic distances and, therefore, still lacks more precise spatial information about the molecule.

The SLATM descriptor involves a two-body term, which is a function of the coordinates and atomic numbers of the constituent atoms, and a three-body term, which includes a van der Waals potential contribution based on the Axilrod–Teller–Muto three-body potential.^46^ The presence of a three-body term and thus the inclusion of van-der-Waals interactions in the molecular descriptor indicate a much more elaborate picture of the impact of the surrounding environment on each atom. Accordingly, SLATM has proven to be a more complex molecular representation that yields better performance in ML models, albeit with increased computational costs for its generation and higher running costs due to larger sizes compared to two-body descriptors like BOB. However, SLATM still only considers neighboring atoms and is therefore not prohibitively expensive, making it suitable for extensive benchmark studies with multiple components where varying parameters and running calculations are required. Although this work primarily focuses on BOB and SLATM as baseline geometric representations, we additionally benchmark selected performance metrics against those obtained using the Smooth Overlap of Atomic Positions (SOAP) descriptor.^58^

##### 2.1.2.2 Electronic representation

The second type of descriptor focuses on the electronic structure features of a given molecular system. Here, our main purpose is to define a reliable and efficient electronic descriptor that does not required large computational overheads rising from highly accurate QM methods (*e.g.*, DFT, coupled-cluster) for which single-point calculations of big conformational datasets of large drug-like molecules are not sustainable for high-throughput studies. Accordingly, the QM properties of molecular conformations are calculated using the semi-empirical third-order DFTB method (DFTB3)^59,60^ supplemented with a treatment of many-body dispersion (MBD) for van der Waals interactions,^61,62^ as it is implemented in the 
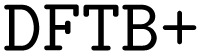
 code.^63^ The versatile performance of DFTB method has already been demonstrated in several works.^50,64–69^ For instance, DFTB simulations were used to gain a molecular understanding of the temperature-gradient degradation of polyethylene and polypropylene and to evaluate the subsequent oxidative upcycling reactions.^64^ DFTB + MBD was also recently used to investigate the relative stability of native states of several proteins in explicit solvation.^65^ Similarly, DFTB has been successfully applied to examine the electrostatic interactions and charge transfer in artificial molecular devices^66,67^ This method has also been extended to investigate excited-state properties, computing electronic transition dipole moments for organic chromophores.^68^

Single-point DFTB3 + MBD calculations were carried out for all molecular systems studied in this work by considering hydrogen correction and the electronic Hamiltonian described by the 
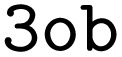
 parameters set.^70^ The QM properties extracted from the DFTB output files are listed in Table 1, and have been divided into global properties (*D*_glob_), molecular orbital energies (*D*_eMO_), and atomic properties (*D*_atom_). To create a standardized representation across the dataset, Mulliken charges arrays (whose dimension depends on the number of atoms in the molecule) are zero-padded to match the array size corresponding to the largest molecular structure in the dataset, so that all properties are included in a fixed-size array. This ensures consistency in the descriptor representation, allowing for effective input to ML models.

**Table 1:** Electronic structure features included in the *D*_QM_ descriptor. These features were calculated using the semi-empirical third-order DFTB method (DFTB3) supplemented with a treatment of many-body dispersion (MBD) for van der Waals interactions. We categorize these properties into three subsets: global, MO energies, and atomic

Subset	Label	Property name	Dim
Global (*D*_glob_)	*E*_Fermi_	Fermi energy	1
	*E*_band_	Band energy	1
	NE	Number of electrons	1
	*E*_H0_	Reference density energy	1
	*E*_scc_	Self-consistent charge energy	1
	*E*_3rd_	Third-order correction energy	1
	*E*_rep_	Repulsion energy	1
	*E*_mbd_	Many-body interaction energy	1
	‖*µ*_TB_‖	Scalar dipole moment	1
	*E* ^TB^_gap_	HOMO–LUMO energy gap	1
MO energies (*D*_eMO_)	*ε*	Molecular orbital energies	8
Atomic (*D*_atom_)	*Q*	Atomic Mulliken charges	N

### 2.2 Benchmark datasets

To understand the effect of considering both geometric and electronic descriptors on the performance of ML regression models, we have first investigated the accuracy in predicting highly accurate physicochemical properties of equilibrium and non-equilibrium conformations of small drug-like molecules contained in QM7-X dataset.^50^ Later, we generated QM structural and property data of molecular conformations associated with SMILES representation of large drug-like molecules extracted from LD_50_ toxicity^51,52^ and lipophilicity datasets.^36^

#### 2.2.1 Physicochemical properties

QM7-X dataset^50^ provides QM-based physicochemical properties for approximately 4.2 M equilibrium and non-equilibrium structures of molecules containing up to seven non-hydrogen atoms (C, N, O, S, and Cl). For equilibrium structures, SMILES strings from the GDB13 database were used to enumerate structural/constitutional isomers and stereoisomers. A diverse set of (*meta*-)stable conformers was then generated and optimized using DFTB3+MBD method. To explore non-equilibrium structures, 100 configurations per molecule were generated by perturbing the stable structure along linear combinations of normal mode coordinates. These perturbations were designed to yield energy differences calculated with DFTB3+MBD that followed a Boltzmann distribution. This approach ensured efficient sampling of critical regions of the potential energy surface near the (*meta*-)stable structures, while including a limited number of high-energy non-equilibrium structures. For each molecular structure, over 40 molecular (global) and atomic (local) properties were calculated at the higher-fidelity DFT-PBE0 hybrid functional supplemented with MBD correction, which has been shown to provide accurate and reliable descriptions of intramolecular and intermolecular degrees of freedom. Accordingly, to better elucidate the predictive capabilities of the descriptors, we have studied the equilibrium and the most distorted structure per molecular conformer, *i.e.*, we have two QM7-X subsets and each of them contains circa 42k structures. The target properties include atomization energy *E*_AT_ and scalar dipole moment *µ*, both ground state properties, as well as the HOMO–LUMO (Highest Occupied Molecular Orbital – Lowest Unoccupied Molecular Orbital) gap *E*_gap_, an intensive property, and the scalar isotropic polarizability *α*, a response quantity derived using the self-consistent screening approach.

#### 2.2.2 Biological responses

The median lethal dose (LD_50_) for oral acute toxicity represents the dose of a substance required to lethally affect 50% of a test population (typically rodents) within a specified exposure period.^51^ This metric serves as an initial assessment of chemical toxicity, aiding in the classification of substances based on their potential acute hazard to human health.^71^ The Therapeutic Data Commons^72^ (TDCommons) platform provides an acute *in vivo* toxicity dataset, originally compiled by Zhu *et al.*^52^ in 2009, which includes the SMILES representations of 7385 compounds and their experimentally determined oral rat LD_50_ values. These values are expressed as the chemical dose per kilogram of body weight, converted to log((mol^−1^ kg^−1^)) values following standard QSAR conventions. The dataset considers chemical compounds containing 2 to 90 non-hydrogen atoms (C, N, O, F, Si, P, S, Cl, Br, and I), and with LD_50_ values ranging from −0.34 to 10.20, providing a broad representation of acute oral toxicity profiles suitable for training predictive models. In an initial screening step, we excluded molecules containing Si atoms from further analysis, as this element is not included in the 3ob SK parameters (see summary of discarded molecules in Table S1 of the SI).

Additionally, we investigated the prediction of lipophilicity in drug-like molecules. This property describes the tendency of a compound to partition into a non-polar lipid matrix rather than an aqueous matrix.^73^ Lipophilicity is strongly correlated with key physicochemical and biochemical properties such as permeability and solubility, which in turn influence drug potency, distribution, and elimination. Consequently, it is frequently measured in QSAR studies to better characterize the pharmacological profiles of drug-like compounds.^74^ The lipophilicity dataset from the MoleculeNet benchmark^36^ contains experimental measurements of the octanol/water distribution coefficient (log *D* at pH 7.4) for 4200 compounds. These compounds contain heavy atom counts (B, C, N, O, F, Cl, P, S, Se, Si, Br, and I) ranging from 7 to 100 and log *D* values spanning −1.5 to 4.5. Eleven compounds were discarded because they contained Si (similar to TDCommons dataset) or B, P, or Se, which were underrepresented in the dataset (see summary of discarded molecules in Table S1 of the SI).

To achieve a more precise modelling and representation of these drug-like molecules for predicting toxicity and lipophilicity, we generated their 3D molecular structures. The initial 3D structures were created using the 
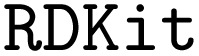
 package, which constructs the graph of heavy atoms based on the connectivity information encoded in SMILES strings, adds hydrogen atoms, and optimizes the resulting geometry using the Merck Molecular Force Field (MMFF). For the next step, we considered only the molecules that were successfully generated and optimized using MMFF. While calculating ground-state energies and geometries *via ab initio* methods would offer greater accuracy, the computational demands are prohibitive. As a practical alternative, we employed the Conformer–Rotamer Ensemble Sampling Tool (CREST),^75,76^ which performs a comprehensive conformational search. CREST plays a crucial role in QUED by efficiently sampling both low- and high-energy conformers with a level of complexity beyond that of well-known conformational search methods based on classical force fields.^77^ This advantage arises from its more accurate treatment of long-range interactions (electrostatics and dispersion) and its ability to incorporate solvent effects. Indeed, CREST integrates the semi-empirical extended tight-binding method GFN2-xTB^78^ with a metadynamics-based search algorithm. We have applied energy (12.0 kcal mol^−1^) and root-mean-square deviation (RMSD) (0.1 Å) thresholds relative to the input structure to determine which geometries are included in the final Conformer–Rotamer Ensemble (CRE). All geometry optimization and conformational search calculations were performed using the GBSA implicit solvent model for water. As a result, 98.5% (7273 unique molecules) of the toxicity dataset and 97.0% (4073 unique molecules) of the lipophilicity dataset were successfully retrieved, yielding approximately 3.6 M and 1.8 M conformers, respectively. We then applied an RMSD-based hierarchical clustering method to refine these extensive sets, selecting ≈ 1.8 M and 618k representative conformers. This clustering approach ensures that the chosen conformers effectively represent the diversity of the explored conformational space. For each target property, the ML regression models were trained using only the conformer with the lowest DFTB3+MBD energy for each unique molecule. The computational costs associated with dataset generation are summarized in Fig. S5 of the SI.

## 3 Results and discussion

### 3.1 Assessing QM descriptors for small molecules

**Fig. 3:**
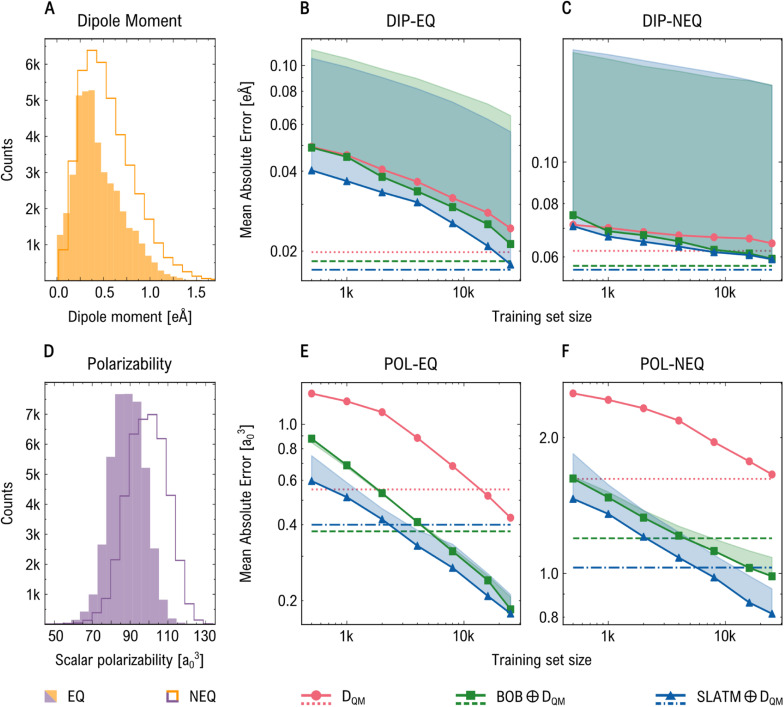
Performance of regression models trained on the QM7-X dataset for predicting DFT-PBE0 dipole moment (DIP) and polarizability (POL). Panels (A) and (D) show property distributions for equilibrium (filled histograms) and non-equilibrium (empty histograms) geometries. Panels (B) and (C) show the learning curves for the prediction of dipole moment of equilibrium (EQ) and non-equilibrium (NEQ) molecular subsets. While panels (E) and (F) show the learning curves for polarizability. We present the mean absolute errors (MAEs) obtained by KRR method using *D*_QM_, BOB ⊕*D*_QM_, and SLATM ⊕*D*_QM_ as solid lines; shaded areas highlight improvements from adding *D*_QM_ to geometric descriptors. Dashed lines indicate performance of XGBoost models trained with 25k samples. Across tasks, combined descriptors consistently outperform their purely geometric counterparts.

We first explore the impact of combining geometric and electronic representations on the performance of ML regression models in predicting both extensive (atomization energy, *E*_AT_, and molecular polarizability, *α*) and intensive (dipole moment, *µ*, and HOMO–LUMO gap, *E*_gap_) physicochemical properties of small drug-like molecules from the QM7-X dataset (see Fig. 3 and S1 of SI). Two molecular subsets were analyzed: one consisting of equilibrium conformations (EQ) and the other of highly distorted non-equilibrium conformations (NEQ), each containing approximately 42k structures. Fig. 3A, D and S1A, D show the distributions of the target properties across both subsets. Our results indicate that combining baseline representations (BOB or SLATM) with electronic descriptors (*D*_QM_), *i.e.*, BOB ⊕*D*_QM_ and SLATM ⊕*D*_QM_, consistently improves prediction accuracy across all regression tasks when using KRR method. Among all models, SLATM ⊕*D*_QM_ yields the highest accuracy. The benefit of including electronic descriptors is particularly significant for the NEQ subset, where the mean absolute error (MAE) is reduced by approximately 60% on average for all properties except *α*. This underscores the difficulty of capturing the relationship between strongly distorted molecular geometries and their properties using geometric features alone. In these cases, electronic descriptors derived from DFTB calculations enhance the connectivity between data points, leading to a more meaningful mapping between the high-dimensional feature space and the target properties. In contrast, for the EQ subset, incorporating electronic descriptors yields a more pronounced improvement for intensive properties than for extensive ones. Overall, the results obtained using the XGBoost method follow similar trends to those from KRR. However, while KRR slightly outperforms XGBoost in predicting extensive properties, XGBoost performs better for intensive properties (see dotted and dashed horizontal lines in Fig. 3 and S1 of SI). Table 2 presents the top results for predicting dipole moments and polarizabilities.

**Table 2:** Summary of the best-performing regression models for predicting molecular physicochemical properties in the QM7-X dataset. Performance is evaluated using mean absolute error (MAE) and the coefficient of determination (*R*^2^) for dipole moment (*µ*) and polarizability (*α*). Results are reported for Kernel Ridge Regression (KRR) and XGBoost models employing different molecular descriptors on both QM7-X subsets (EQ and NEQ)

Target	Dataset	Regression method	Descriptor	MAE	*R* ^2^
Dipole moment	EQ	KRR	SLATM ⊕*D*_QM_	0.018	0.987
			*D*_QM_	0.024	0.979
		XGBoost	SLATM ⊕*D*_QM_	**0.017**	**0.988**
			*D*_QM_	0.023	0.982
	NEQ	KRR	SLATM ⊕*D*_QM_	0.059	0.924
			*D*_QM_	0.065	0.911
		XGBoost	SLATM ⊕*D*_QM_	**0.056**	**0.933**
			*D*_QM_	0.062	0.919
Polarizability	EQ	KRR	SLATM ⊕*D*_QM_	**0.178**	**0.999**
			*D*_QM_	0.426	0.994
		XGBoost	BOB ⊕*D*_QM_	0.377	0.995
			*D*_QM_	0.552	0.988
	NEQ	KRR	SLATM ⊕*D*_QM_	**0.814**	**0.988**
			*D*_QM_	1.657	0.958
		XGBoost	SLATM ⊕*D*_QM_	1.030	0.984
			*D*_QM_	1.620	0.957

We now turn to the prediction of *µ*, which serves as a clear example of how QM-derived features can enhance molecular representations for predicting intensive properties (results for *E*_gap_ can be found in Tables S4, S5 and Fig. S1 of SI). As shown in Fig. 3B and C, *D*_QM_ significantly outperforms the purely geometric descriptors BOB and SLATM, achieving MAE values of 0.024 and 0.064 eÅ for EQ and NEQ subsets, respectively, when using KRR models trained on 25k samples. Combining geometric and electronic descriptors further improves the prediction accuracy, yielding MAE values of 0.018 and 0.059 eÅ with the SLATM ⊕*D*_QM_. Similarly, XGBoost models confirm the advantage of incorporating QM-derived information: while *D*_QM_ alone achieves an MAE of 0.023 eÅ, adding two-body interactions reduces this to 0.018 eÅ, and including three-body features further improves it to 0.017 eÅ, demonstrating consistently improved performance for *µ* prediction of EQ subset (similar trend is also found for NEQ subset). In contrast, for extensive properties, purely geometric descriptors generally outperform *D*_QM_ across learning curves. When combining both types of descriptors, MAE values either remain unchanged or improve only marginally relative to the geometric descriptor alone. Notable improvements are mainly observed at larger training set sizes (16k and 25k), particularly for the SLATM representation. For instance, in predicting *α* for EQ subset (see Fig. 3E), SLATM shows a consistent improvement of approximately 15% when combined with *D*_QM_, achieving an MAE value of 0.18 *a*_0_^3^. For NEQ subset (see Fig. 3F), both BOB and SLATM representations show enhanced performance when combined with *D*_QM_, with MAEs decreasing from 1.08 to 0.99 *a*_0_^3^ and from 0.92 to 0.81 *a*_0_^3^, respectively. Unlike *µ* prediction, XGBoost models generally yield higher errors than KRR models for extensive properties. Interestingly, for EQ subset, the best performance is achieved by the combination BOB ⊕*D*_QM_ (MAE = 0.377 *a*_0_^3^), diverging from the trend observed in other tasks, where SLATM ⊕*D*_QM_ typically performs better. A similar behavior is seen in the prediction of *E*_AT_ (see Tables S4, S5 and Fig. S1 of SI). Although the addition of *D*_QM_ does not consistently outperform geometric descriptors for EQ subset, its value becomes more evident for NEQ subset. These findings are further supported by KRR models employing delta learning (see Table S6 of SI) and the XGBoost models using SOAP descriptor (see Table S10 of SI), which show consistent improvements in predictive accuracy when incorporating *D*_QM_.

#### 3.1.1 Interpreting descriptor performance

##### 3.1.1.1 Descriptor components

**Fig. 4:**
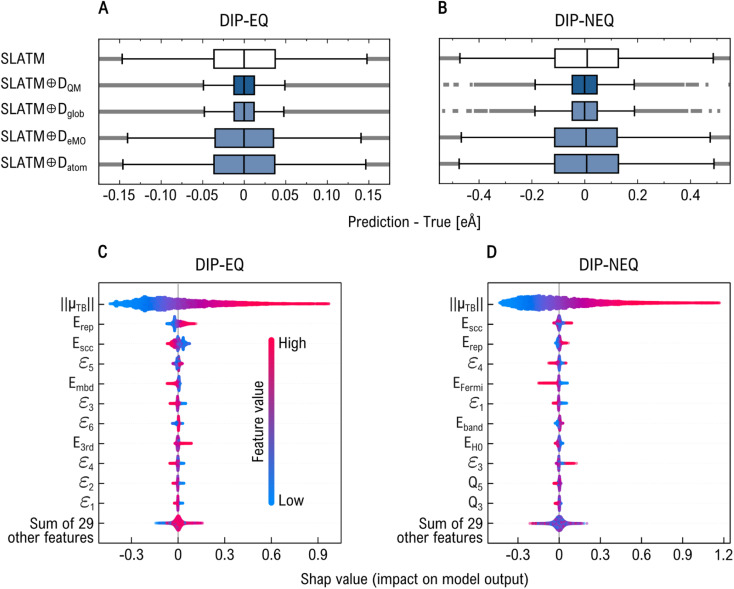
Evaluation of regression models to predict DFT-PBE0 dipole moment when combining SLATM with subsets of the *D*_QM_ descriptor. Panels (A) and (B) show the distribution of residuals (prediction – true) for KRR models on equilibrium (EQ) and non-equilibrium (NEQ) subsets, respectively, using global (*D*_glob_), MO energy (*D*_eMO_), and atomic (*D*_atom_) components. Panels (C) and (D) display SHAP (which stands for ‘SHapley Additive exPlanations”) value distributions, ranking features by relevance in the predictions made by XGBoost models using only *D*_QM_ descriptor. Key contributors include the norm of the tight-binding dipole moment (‖*µ*_TB_‖), DFTB energy terms, MO energies (*ε*_*i*_), and Mulliken charges (*Q*_*j*_).

**Fig. 5:**
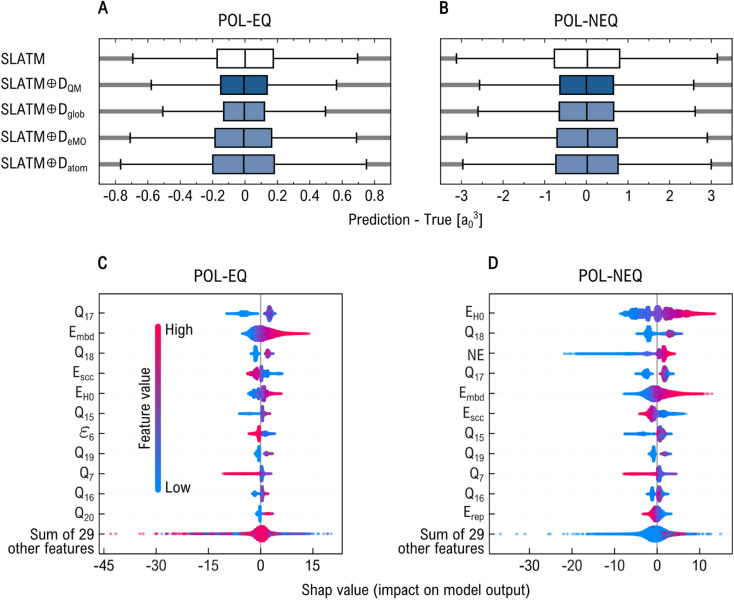
Evaluation of regression models to predict DFT-PBE0 molecular polarizability when combining SLATM with subsets of the *D*_QM_ descriptor. Panels (A) and (B) show the distribution of residuals (prediction – true) for KRR models on equilibrium (EQ) and non-equilibrium (NEQ) subsets, respectively, using global (*D*_glob_), MO energy (*D*_eMO_), and atomic (*D*_atom_) components. Panels (C) and (D) display SHAP (which stands for ‘SHapley Additive exPlanations”) value distributions, ranking features by relevance in the predictions made by XGBoost models using only *D*_QM_ descriptor. Key contributors include many-body dispersion (MBD) energy and Mulliken charges (*Q*_*j*_).

To determine which components of the *D*_QM_ descriptor most significantly enhance model performance, we partitioned it into three subsets: global (*D*_glob_), MO energies (*D*_eMO_), and atomic (*D*_atom_) properties. A detailed list of these properties is provided in Table 1. We assessed the performance of the geometric descriptor SLATM both “pure” and in combination with each property subset, *i.e.*, SLATM ⊕*D*_glob_, SLATM ⊕*D*_eMO_, and SLATM ⊕*D*_atom_, as well as with the full QM descriptor (SLATM ⊕*D*_QM_). The results from the KRR models trained on 16k samples are shown in Fig. 4 and 5 for the prediction of *µ* and *α*, respectively.

Optimal performance in physicochemical property prediction was generally achieved by combining the SLATM representation with the full electronic descriptor. However, for EQ subset, more accurate estimates of *µ* and *α*—20.5 × 10^−3^ eÅ and 0.18 *a*_0_^3^, respectively—were obtained using SLATM ⊕*D*_glob_, compared to 20.9 × 10^−3^ eÅ and 0.21 *a*_0_^3^ for SLATM ⊕*D*_QM_. As shown in the boxplots of Fig. 4A and 5A, both models produce comparable error ranges, with SLATM ⊕*D*_glob_ exhibiting a slightly narrower error distribution. For NEQ subset, the global feature set also leads to substantial performance improvements when combined with SLATM, ranking second only to SLATM ⊕*D*_QM_. We attribute this improvement to the inclusion of the TB dipole moment and many-body dispersion energy in *D*_glob_, which show strong correlations with the reference DFT-PBE0 *µ* (*ρ*(*µ*, *µ*_TB_) = 0.94) and *α* (*ρ*(*α*, *E*_mbd_) = −0.61), respectively (see other *ρ* values in Fig. S3 of SI). Interestingly, even though *D*_glob_ includes the HOMO–LUMO energy gap at the DFTB3 level (*E*^TB^_gap_)–a property that shows only moderate correlation with the DFT-PBE0 energy gap–SLATM ⊕*D*_eMO_ outperforms all other models for predicting *E*_gap_. On the other hand, incorporating Mulliken charges (*Q*) generally degrades performance and appears to introduce noise, even though the target properties are related to the spatial distribution of charge. In most tasks, the SLATM ⊕D_atom_ model performs worse than SLATM alone, likely due to the weak correlation between *Q* values and the target properties (see Fig. S3 of SI). A similar trend is observed for *D*_eMO_ in the prediction of *µ* and *α*, which may also be correlated to the overall weak correlation of these properties with MO energies. However, SLATM ⊕*D*_eMO_ model does outperform SLATM for predicting *E*_AT_ and *E*_gap_, being the effect more remarkable for NEQ subset (see Fig. S2 of SI). These findings suggest that, while strong physical correlations with the target properties are important, model performance also depends critically on how electronic structure information is represented and integrated with geometric descriptors.

##### 3.1.1.2 SHapley additive exPlanations (SHAP)

Tree-based predictive ML models, such as XGBoost, greatly benefit from being coupled with interpretability tools like the SHAP method.^53^ Rooted in cooperative game theory, SHAP leverages Shapley values to quantify the contribution of each input feature to an individual prediction. In the context of our feature-based ML model, SHAP values estimate how each feature influences the deviation of a specific prediction from the expected output of the model. This enables a transparent interpretation of the learned relationships, revealing both the relative importance of features and how they interact to shape predicted outcomes. Fig. 4 and 5 present beeswarm plots that summarize the distribution of SHAP values for the most influential features in each property prediction task, based solely on *D*_QM_. In these plots, features are ordered by importance (top to bottom), and their SHAP values are shown along the *x*-axis. A positive SHAP value indicates that the feature pushes the prediction higher, while a negative value suggests it drives the prediction lower. The color gradient encodes the feature values: red denotes high values, and blue denotes low values.

Fig. 4C and D show that *µ*_TB_ is the most influential feature in predicting the DFT-PBE0 dipole moment (*µ*). This result is expected, as both quantities represent the same physical observable, albeit computed using different QM methods. In the EQ subset, we observe that higher values of the DFTB repulsion energy (*E*_rep_) and the third-order correction energy (*E*_3rd_) are associated with larger dipole moments. In contrast, higher values of the self-consistent charge energy (*E*_scc_) and many-body dispersion energy (*E*_mbd_) tend to reduce the predicted *µ*. Notably, the TB-derived MO energies also rank among the top ten relevant features. A similar pattern is observed for the NEQ subset, where Mulliken charges (*Q*) also emerge as important contributors to the predictive performance. SHAP analysis for *α* predictions reveals a slightly different hierarchy of feature importance between EQ and NEQ subsets (see Fig. 5C and D). Overall, low values of Mulliken charges tend to negatively impact the predicted polarizability, with the notable exception of *Q*_7_, which deviates from this trend. We identify *E*_mbd_ as a key feature in this regression task: low values are linked to smaller polarizabilities, while higher values correlate with increased polarizability. This is consistent with the moderate negative correlation between *E*_mbd_ and *α* (*ρ*(*E*_mbd_, *α*) = −0.61). Interestingly, the reference DFTB energy (*E*_H0_) also plays a significant role, likely due to its influence on the electron density distribution and its response to perturbations. In NEQ subset, the number of electrons contributes positively to the prediction, whereas it does not rank among the top 11 features for the EQ subset. Surprisingly, for EQ subset, the sixth MO energy *ε*_6_ gains more relevance, indicating that different structural regimes may be governed by distinct sets of driving QM features.

### 3.2 Predicting biological responses of large molecules

We now examine QUED performance to predict biological endpoints: toxicity and lipophilicity. To this end, we develop ML regression models using chemically diverse sets of large drug-like molecules from the TDCommons-LD_50_ dataset and the MoleculeNet-Lipophilicity dataset. These models were trained on the lowest-energy geometries (as determined by DFTB3+MBD) for each unique molecule.

**Fig. 6:**
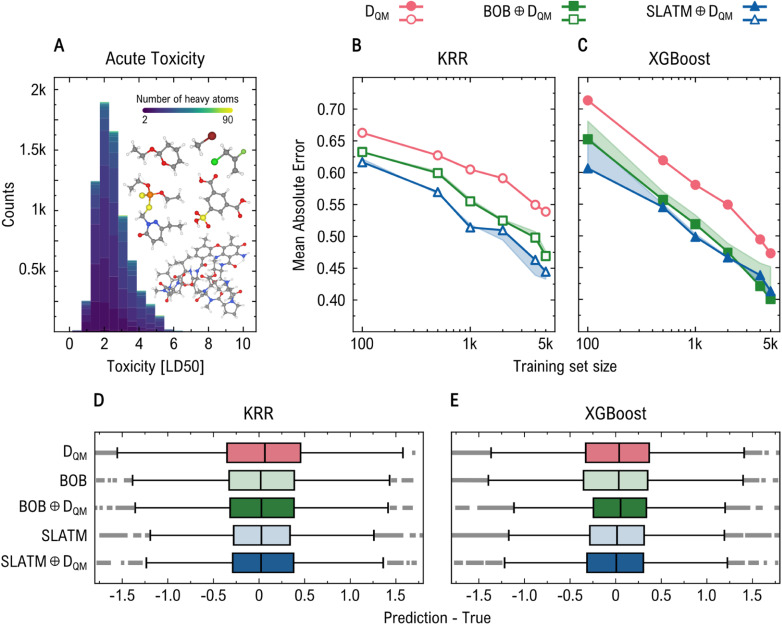
Prediction of acute toxicity (LD_50_) of large drug-like molecules from TDCommons-LD_50_ dataset.^72^ Panel (A) shows the LD_50_ distribution, colored by the number of non-hydrogen atoms. Panels (B) and (C) present learning curves for KRR and XGBoost models, respectively, using *D*_QM_, BOB ⊕*D*_QM_, and SLATM ⊕*D*_QM_. Shaded areas highlight improvements from adding *D*_QM_ to geometric descriptors. Panels (D) and (E) display the corresponding residual distributions (prediction – true). In this task, adding *D*_QM_ improves the performance of BOB but not SLATM, highlighting that the benefit of electronic information is not only task-specific but also descriptor-dependent. For these calculations, we used only the lowest-energy conformation of each unique molecule in the TDCommons-LD_50_ dataset.

The distribution of LD_50_ values in this subset is shown in Fig. 6A. Fig. 6B and C present the learning curves for *D*_QM_ and BOB ⊕*D*_QM_ and SLATM ⊕*D*_QM_, using the KRR and XGBoost methods, respectively. In this task, XGBoost models consistently outperformed their KRR counterparts. This performance difference between XGBoost and KRR models is further illustrated in Fig. 6D and E, which display box plots of residuals (predicted minus true toxicity values) for different descriptor combinations. The KRR residuals show a broader spread, indicating higher prediction variance and reduced precision, whereas the XGBoost residuals are more tightly clustered around zero. Under KRR, the inclusion of geometric information improves the performance of the electronic descriptor. For instance, *D*_QM_ alone yields an MAE of 0.539, which decreases to 0.469 and 0.445 when combined with BOB and SLATM, respectively. Interestingly, the combination SLATM ⊕*D*_QM_ slightly underperforms pure SLATM, which achieves an MAE of 0.433—suggesting that in this case, the addition of *D*_QM_ may not be beneficial. In contrast, under XGBoost, *D*_QM_ achieves an MAE of 0.473. BOB shows improved performance when combined with *D*_QM_, achieving the best overall result with an MAE of 0.400—an improvement over the 0.451 obtained with pure BOB. In contrast, SLATM does not benefit from this addition: pure SLATM reaches an MAE of 0.403, slightly better than SLATM ⊕*D*_QM_, which yields 0.413.

On the other side, geometric descriptors show no relevant improvement when combined with *D*_QM_ for lipophilicity prediction (see Fig. S4 of SI). Alike toxicity results, XGBoost consistently outperforms KRR, *e.g.*, pure *D*_QM_ yields MAEs of 0.784 and 0.617 with KRR and XGBoost, respectively. For KRR, the best performance is achieved with SLATM ⊕*D*_QM_ (MAE = 0.476), followed closely by pure SLATM (MAE = 0.480). For XGBoost, the best model overall is pure SLATM (MAE = 0.418), with performance slightly reduced upon inclusion of *D*_QM_ (MAE = 0.432).

Table 3 summarizes the best results for predicting toxicity and lipophilicity. Our XGBoost model using BOB ⊕*D*_QM_ achieves an MAE of 0.400 on the TDCommons-LD_50_ dataset, surpassing previous state-of-the-art approaches. These include 2D graph neural networks,^79^ which achieved an MAE of 0.45 when considering only the top 5% most confident predictions; equivariant transformers^38^ with an MAE of 0.653; and fingerprint-based surrogate models,^80,81^ reporting MAEs of 0.497 and an RMSE of 0.697, respectively. Furthermore, combining pure SLATM with XGBoost yields the best predictive accuracy for lipophilicity, with an RMSE of 0.567. This result surpasses the benchmark set by MoleculeNet using Extended-Connectivity Fingerprints with XGBoost (0.799) and is comparable to their results using graph-convolutional methods (0.655).^36^ While more complex state-of-the-art architectures reach comparable errors, such as convolutional neural networks trained on augmented SMILES representations^82^ (RMSE = 0.593), graph neural networks and multitask learning^83^ (RMSE = 0.537), 3D molecular representation learning framework Uni-Mol^84^ (RMSE = 0.603), and nested connected hierarchical GNN DenseNGN^85^ (MAE = 0.351), our approach remains competitive due to its simplicity and computational efficiency. Notice that regression models trained with the SOAP descriptor using XGBoost (see Table S10 in the SI) and with the state-of-the-art equivariant neural network MACE^86^ (see Table S11 in the SI) exhibited lower performance compared to the top-performing models summarized in Table 3.

**Table 3:** Summary of the best-performing regression models for predicting biological responses. Reported metrics include mean absolute error (MAE), root mean squared error (RMSE), and coefficient of determination (*R*^2^) for toxicity (LD_50_) and lipophilicity (log *D*) prediction using Kernel Ridge Regression (KRR) and XGBoost methods with different molecular descriptors

Target	Regression method	Descriptor	MAE	RMSE	*R* ^2^
Toxicity	KRR	SLATM	0.433	0.606	0.595
		*D*_QM_	0.539	0.719	0.430
	XGBoost	BOB ⊕*D*_QM_	**0.400**	**0.571**	**0.661**
		*D*_QM_	0.473	0.637	0.572
Lipophilicity	KRR	SLATM ⊕*D*_QM_	0.476	0.661	0.643
		*D*_QM_	0.784	0.991	0.339
	XGBoost	SLATM	**0.418**	**0.567**	**0.752**
		*D*_QM_	0.617	0.813	0.519

#### 3.2.1 Descriptor components

Following the approach used in the previous section, we combined BOB and SLATM with subsets of *D*_QM_ to assess which electronic properties most strongly influence toxicity and lipophilicity prediction using KRR models (see Tables S7 and S8 of SI). Although the features in *D*_QM_ show only weak correlation with LD_50_ values (see Fig. S3 of SI) and the complete descriptor does not improve SLATM performance, we find that the combinations SLATM ⊕*D*_glob_ and SLATM ⊕*D*_eMO_ slightly reduce the MAE to 0.426 and 0.429, respectively (see Table S7 of SI). In contrast, SLATM ⊕*D*_atom_ leads to a higher error of 0.452. This trend is not observed for BOB: while BOB ⊕*D*_glob_ and BOB ⊕*D*_eMO_ only marginally increase the MAE from 0.476 to 0.479 and 0.480, respectively, BOB ⊕*D*_atom_ achieves the same MAE as the pure geometric descriptor.

The correlation between lipophilicity and the properties in the electronic descriptor is even weaker compared to toxicity (see *ρ* values in Fig. S3 of SI). Consequently, adding *D*_eMO_ or *D*_atom_ to the pure BOB descriptor results in larger MAEs, *i.e.*, 0.585 and 0.593, respectively (see Table S8 of SI). In contrast, SLATM ⊕*D*_eMO_ and SLATM ⊕*D*_atom_ achieve slightly better performances (0.478 and 0.479) than pure SLATM. For both geometric descriptors, the inclusion of *D*_glob_ has no significant impact on performance. These results indicate that the benefit of incorporating QM features depends on the base geometric descriptor and target biological response; hence, the integration should be adapted to their specific characteristics.

#### 3.2.2 SHapley additive exPlanations (SHAP)

**Fig. 7:**
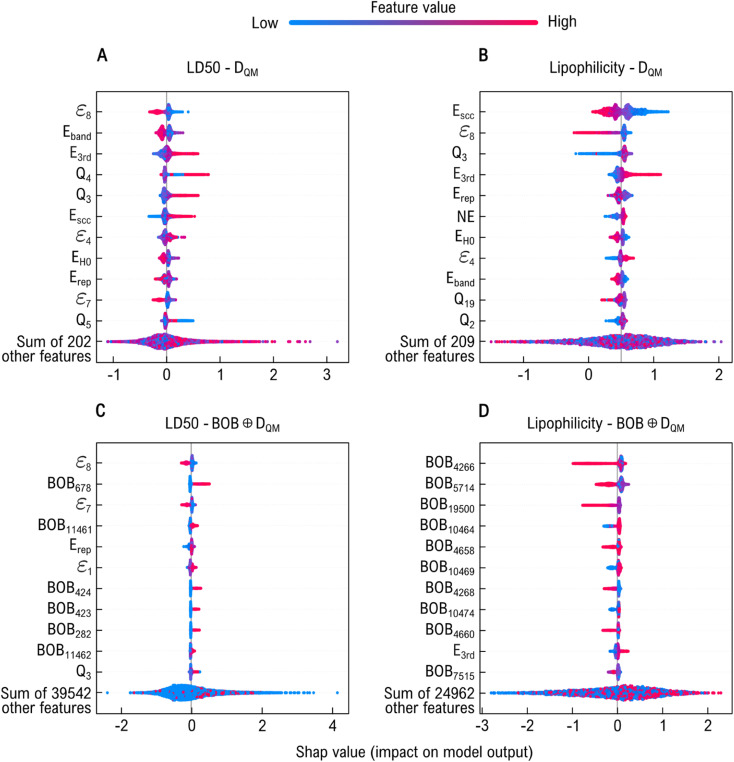
Feature importance analysis for acute toxicity (LD_50_) and lipophilicity prediction. Panels (A) and (B) show SHAP value distributions for XGBoost models trained with *D*_QM_, while panels (C) and (D) show the corresponding distributions for BOB ⊕*D*_QM_. Geometric features strongly shape model predictions, providing fine-grained distinctions that enhance clustering and predictive performance, and generally dominate over electronic descriptors in the combined representations.

We perform a SHAP analysis on the *D*_QM_ and BOB ⊕*D*_QM_ XGBoost models to evaluate the relevance of QM and BOB features in learning acute toxicity and lipophilicity (see Fig. 7). Fig. 7A and B show that all subsets of *D*_QM_ (*i.e.*, global, electronic, and atomic) contribute meaningfully to both prediction tasks. Specifically, high Mulliken charge values appear to positively influence predicted toxicity, whereas tight-binding eigenvalues (*ε*_*i*_) show an inverse relationship. Based on previous studies^29,33^ and our own work, we find that molecular orbital energies (or *ε*_*i*_ energies) are key descriptors for toxicity prediction, as they quantify molecular interactions between a chemical and its site of toxic action.^87^ In particular, the LUMO has been reported to show a direct correlation with intravenous LD_50_ values^88^ and has been identified as the frontier orbital involved in drug-target interactions. DFTB energy contributions also rank among the most relevant features, although their influence on toxicity prediction varies in direction. Similarly, for lipophilicity, energetic components contribute strongly to its prediction, whereas low Mulliken charge (*Q*_*i*_) values of the third atomic component reduce predictive accuracy. Moreover, lipophilicity (and permeability) can be related not only to the molecular charge distribution (represented as *Q*_*i*_) but also to the delocalization and distortion of the electronic cloud within the molecule.^32,89,90^ These electronic effects are captured by the *E*_3rd_ DFTB energy component, which depends on atomic charge fluctuations.^63^ When analyzing the results for BOB ⊕*D*_QM_ in Fig. 7C and D, we find that geometry-based features (BOB_k_) strongly influence the model output. In general, higher-order geometric feature values exert a greater impact on predicting both biological responses, which shifts some QM features to lower ranks in the SHAP analysis. This effect is especially pronounced for lipophilicity. Still, tight-binding eigenvalues remain among the top contributors to toxicity prediction, underscoring their consistent relevance. Compared to the broad spread of SHAP values in *D*_QM_, where the model relies heavily on a few dominant quantum features, BOB ⊕*D*_QM_ shows a more uniform small contribution across features. This indicates that the geometrical information provided by BOB adds fine-grained distinctions for individual data points that help the model form more precise clusters and improve predictive performance.

## 4 Conclusions

In this work, we introduced the “QUantum Electronic Descriptor” (QUED) framework, which integrates both structural and electronic molecular information to develop ML regression models for physicochemical and biological property prediction. Central to QUED was the definition of a QM descriptor derived from molecular and atomic properties computed using the semi-empirical DFTB3 method supplemented with a many-body dispersion (MBD) treatment for van der Waals interactions. Indeed, to form comprehensive molecular representations, we combined this QM descriptor with computationally inexpensive geometric descriptors that capture two-body and three-body interatomic interactions, such as BOB and SLATM. As a proof of concept, we validated QUED performance by using two molecular subsets of the QM7-X dataset, which includes both equilibrium and non-equilibrium conformations of small drug-like molecules. The results demonstrated that incorporating electronic structure information significantly improves the accuracy of ML models in predicting physicochemical properties compared to only considering geometric features. In particular, combining SLATM with *D*_QM_ led to a notable accuracy improvement, especially for highly distorted molecular structures. For QM7-X molecules, XGBoost models followed similar trends to those obtained by KRR models trained using 
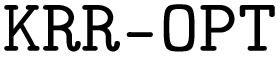
 toolbox. However, while KRR slightly outperforms XGBoost in predicting extensive properties, XGBoost performs better for intensive properties. Moreover, a detailed analysis combining property subsets with SHAP method revealed that certain electronic features are more relevant for specific target physicochemical properties, *e.g.*, global properties play a more crucial role than MO energies or atomic charges in predicting *µ*, whereas atomic charges and DFTB energy components are more important for predicting *α*.

QUED framework was also evaluated on the TDCommons-LD_50_ and MoleculeNet-Lipophilicity datasets to predict the toxicity levels and lipophilicity of larger and more chemically diverse drug-like molecules, respectively. Here, the benefits and insights of using QM descriptors for biological property prediction were more nuanced. SHAP analysis also confirmed that DFTB properties, such as MO energies and energy components, play a central role in these performance gains, with BOB ⊕*D*_QM_ combined with XGBoost yielding the best performance for toxicity prediction. Overall, our findings highlight the importance of incorporating electronic structure data into ML workflows to enhance the reliability and interpretability of the predictive models. While geometric descriptors capture spatial patterns effectively, they may miss subtle electronic effects that are critical for accurately modelling complex molecular properties. That said, the computational demands associated with QM descriptor generation—even when using semi-empirical methods—can be a bottleneck for high-throughput workflows. To address this trade-off between descriptor complexity and computational efficiency, future research should explore strategies to optimize both aspects. One promising direction is the integration of ML-accelerated electronic structure methods,^91–93^ which can significantly reduce the time required to compute QM descriptors. Additionally, ML-enhanced DFTB approaches offer a way to improve the accuracy of QM properties without significantly increasing computational cost.^94,95^ Within the QUED framework, an analysis of computational time for each step revealed that conformational sampling with CREST represents the most time-consuming component (see Fig. S5 in the SI). In this context, generative AI models could aid conformational exploration, thereby increasing both the diversity and quality of biological datasets.^96–98^ Hence, we expect that the QUED framework can be extended to predict a wide range of biological endpoints, such as ADMET properties (beyond toxicity and lipophilicity), and protein–ligand interactions, further demonstrating the versatility and impact of integrating electronic structure information into molecular ML approaches.

## Author contributions

The work was initially conceived by AH and LMS, and designed with contributions from AT. AK developed the KRR–OPT toolbox. AH and LMS generated the quantum-mechanical datasets, trained the regression models, and analyzed their performance. AT and LMS supervised and revised all stages of the work. AH and LMS drafted the original manuscript. All authors discussed the results and contributed to the final manuscript.

## Conflicts of interest

There are no conflicts to declare.

## Supplementary Material

DD-005-D5DD00411J-s001

## Data Availability

All datasets, source code, running examples, and trained ML models presented in this work are available in the QUED GitHub repository (https://github.com/lmedranos/QUED) and are also publicly available on Zenodo under the DOI https://doi.org/10.5281/zenodo.17106019. Supplementary information (SI) is available. See DOI: https://doi.org/10.1039/d5dd00411j.
