# Supplementary material for: Assessing the performance of quantum-mechanical descriptors in physicochemical and biological property prediction
Source: Digit Discov. 2026 Jan 19;5(2):803–18. doi: 10.1039/d5dd00411j (PMC12820757; doi:10.1039/d5dd00411j)
Supplement: DD-005-D5DD00411J-s001 [file DD-005-D5DD00411J-s001.pdf]

Supplementary Information (SI) for Digital Discovery.
This journal is © The Royal Society of Chemistry 2026

Supplementary Information (SI) for:

Assessing the performance of quantum-mechanical descriptors in physicochemical and biological property prediction

Alejandra Hinostroza Caldas[a], **Artem Kokorin**[b], **Alexandre Tkatchenko**[b*], and **Leonardo Medrano Sandonas**[b†*]

[a] *Universidad Nacional de Ingeniería, Av. Túpac Amaru 210, Rímac, Lima 15333, Peru.*

[b] *Department of Physics and Materials Science, University of Luxembourg, L-1511 Luxembourg City, Luxembourg.*

[*] Corresponding authors: Alexandre Tkatchenko (`alexandre.tkatchenko@uni.lu`) and Leonardo Medrano Sandonas (`leonardo.medrano@tu-dresden.de`)

[†] Present address: Institute for Materials Science and Max Bergmann Center of Biomaterials, TUD Dresden University of Technology, 01062 Dresden, Germany.

1 Dataset preparation

Table S1 lists the initial, discarded, and final numbers of molecules for the benchmark datasets QM7-X, TDCommons-LD50, and MoleculeNet-Lipophilicity. Molecules in the NEQ subset of QM7-X were discarded due to unsuccessful completion of DFTB calculations required to obtain QM features. In the Toxicity and Lipophilicity datasets, samples were discarded based on chemical composition criteria and failures in generating initial molecular structures using RDKit, as described in the main text.

Table S1 Specification of the number of conformers considered in each dataset.

**Dataset**	**Initial Number of Molecules**	**Number of Discarded Molecules**	**Final Number of Conformers**
QM7-X (eq)	41,537	0	41,537
QM7-X (neq)	41,537	2	41,535
Toxicity	7,385	112 (=1.5%)	7,273
Lipophilicity	4,200	127 (=3.0%)	4,073

2 Hyperparameter optimization via KRR-OPT

For all models and datasets, the number of training iterations was scaled with the size of the training set to ensure manageable computational cost (see Table S2). Each dataset was partitioned into training, validation, and test subsets. The validation set was used solely for hyperparameter selection, and all final performance metrics reported in this manuscript were evaluated on the test set. For instance, in the QM7-X dataset ($\approx$41k structures), using 25k samples for training results in 5k validation samples and approximately 11k test samples.

Table S2 Training and validation set sizes for the development of Kernel Ridge Regression (KRR) models across all benchmark datasets, including adjusted iteration counts used for hyperparameter optimization.

Dataset	Training points	Validation points	Iterations
QM7-X	500	2,000	16
	1,000	2,000	12
	2,000	2,000	8
	4,000	4,000	4
	8,000	4,000	2
	16,000	4,000	1
	25,000	5,000	1
Toxicity	100	800	32
	500	2,000	16
	1,000	2,000	12
	2,000	2,000	8
	4,000	2,000	8
	5,000	1,250	4
Lipophilicity	100	800	32
	500	2,000	16
	1,000	2,000	12
	2,000	1,600	8
	3,000	600	8

3 Hyperparameter search range for XGBoost

Table S3 summarizes the hyperparameter space explored during optimization. For model training, the dataset was split into training and testing subsets. For physicochemical property prediction on the QM7-X dataset, the models were trained using 25k samples. For toxicity and lipophilicity prediction, the number of training points specified in Table S2 was used to construct the learning curves.

Table S3 XGBoost Hyperparameter Search Space

Parameter	Type	Search Range / Values
λ	Log-uniform	$[10^{-3}, 10]$
α	Log-uniform	$[10^{-3}, 10]$
colsample_bytree	Categorical	$\{0.1, 0.2, \ldots, 1.0\}$
subsample	Categorical	$\{0.1, 0.2, \ldots, 1.0\}$
learning_rate	Categorical	$\{0.008, 0.010, 0.012, 0.014, 0.016, 0.018, 0.020\}$
n_estimators	Categorical	$\{200, 300, 400, 500, 600, 800, 1000, 1500, 2000, 3000, 4000\}$
max_depth	Categorical	$\{5, 7, 9, 11, 13, 15, 17, 20\}$
min_child_weight	Integer	[1, 300]
random_state	Fixed	20240815

4 Prediction of atomization energy and HOMO-LUMO gap for QM7-X molecules

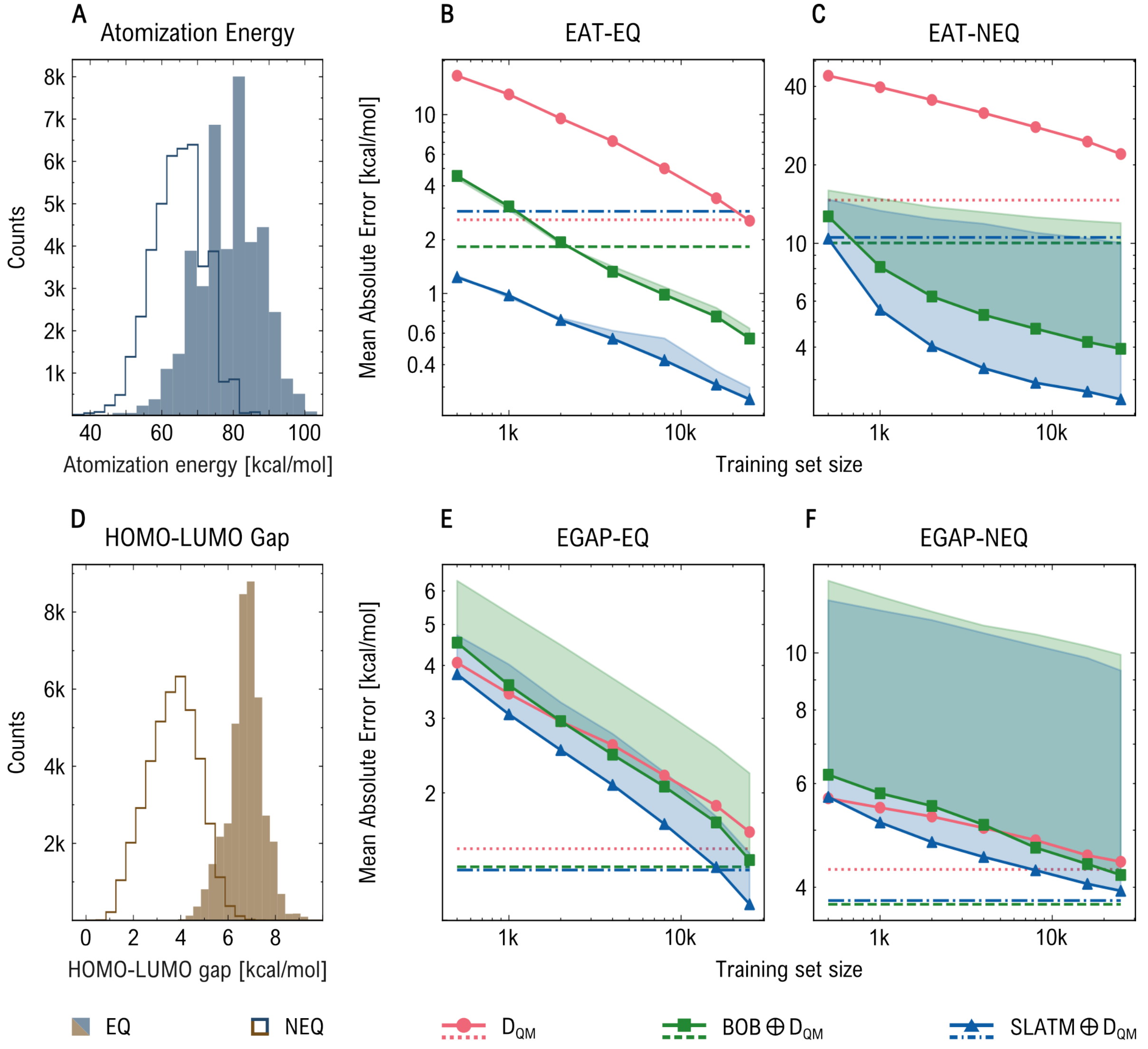

Fig. S1 Performance of models trained on the QM7-X dataset for predicting atomization energies and HOMO–LUMO gaps. Mean absolute errors (MAEs) of Kernel Ridge Regression (KRR) with D_{QM}, BOB $\oplus$ D_{QM}, and SLATM $\oplus$ D_{QM} are shown as solid lines, with shaded regions indicating the improvement gained by adding D_{QM} to geometric descriptors. Dashed lines indicate XGBoost performance trained with 25k samples. Panels **A** and **D** display property distributions for equilibrium (filled) and non-equilibrium (step) geometries. Panels **B** and **C** present atomization energy learning curves, while panels **E** and **F** show HOMO–LUMO gap learning curves.

Target	Set	Metric	D_{QM}	BOB	BOB $\oplus$ D_{QM}	SLATM	SLATM $\oplus$ D_{QM}
EAT	EQ	MAE	2.550	0.638	0.560	0.297	**0.256**
		R2 score	0.999	1.000	1.000	1.000	1.000
	NEQ	MAE	22.004	11.963	3.940	10.046	**2.522**
		R2 score	0.970	0.992	0.999	0.994	0.999
EGAP	EQ	MAE	1.616	2.225	1.387	1.440	**1.090**
		R2 score	0.982	0.964	0.986	0.982	0.991
	NEQ	MAE	4.420	9.928	4.197	9.335	**3.944**
		R2 score	0.942	0.725	0.948	0.754	0.953
POL	EQ	MAE	0.426	0.207	0.185	0.211	**0.178**
		R2 score	0.994	0.999	0.999	0.999	0.999
	NEQ	MAE	1.657	1.084	0.985	0.924	**0.814**
		R2 score	0.958	0.985	0.982	0.987	0.988
DIP	EQ	MAE	0.024	0.065	0.021	0.056	**0.018**
		R2 score	0.979	0.864	0.983	0.877	0.987
	NEQ	MAE	0.065	0.151	0.059	0.151	**0.059**
		R2 score	0.911	0.513	0.925	0.519	0.924

Table S4 Mean absolute error (MAE) and R2 score values for direct learning of target properties via Kernel Ridge Regression. EAT: atomization energy [kcal/mol]; EGAP: HOMO–LUMO gap [kcal/mol]; POL: polarizability [a_0^3]; DIP: dipole moment [eÅ].

Target	Set	Metric	D_{QM}	BOB $\oplus$ D_{QM}	SLATM $\oplus$ D_{QM}
EAT	EQ	MAE	2.576	**1.822**	2.875
		R2 score	0.999	0.999	0.999
	NEQ	MAE	14.605	**10.004**	10.511
		R2 score	0.985	0.993	0.993
EGAP	EQ	MAE	1.472	1.333	**1.311**
		R2 score	0.984	0.988	0.988
	NEQ	MAE	4.278	**3.732**	3.789
		R2 score	0.947	0.958	0.957
POL	EQ	MAE	0.552	**0.377**	0.403
		R2 score	0.988	0.995	0.995
	NEQ	MAE	1.620	1.196	**1.030**
		R2 score	0.957	0.975	0.984
DIP	EQ	MAE	0.023	0.018	**0.017**
		R2 score	0.982	0.986	0.988
	NEQ	MAE	0.062	0.057	**0.056**
		R2 score	0.919	0.933	0.933

Table S 5 Mean absolute error (MAE) and R2 score values for direct learning of target properties via XGBoost. EAT: atomization energy [kcal/mol]; EGAP: HOMO–LUMO gap [kcal/mol]; POL: polarizability [a_0^3]; DIP: dipole moment [eÅ].

Target	Set	Metric	D_{QM}	BOB	BOB $\oplus$ D_{QM}	SLATM	SLATM $\oplus$ D_{QM}
EAT	EQ	MAE	0.921	0.417	0.372	0.243	**0.241**
		R2 score	1.000	1.000	1.000	1.000	1.000
	NEQ	MAE	7.178	3.733	3.091	2.913	**2.487**
		R2 score	0.997	0.999	0.999	0.999	**1.000**
EGAP	EQ	MAE	1.812	2.712	1.411	1.575	**1.066**
		R2 score	0.978	0.945	0.986	0.979	**0.991**
	NEQ	MAE	4.564	7.470	4.234	6.557	**3.886**
		R2 score	0.939	0.767	0.947	0.800	**0.954**

Table S6 Mean absolute error (MAE) and R2 score values for delta learning of target properties via Kernel Ridge Regression models trained on 25k samples. EAT: atomization energy [kcal/mol]; EGAP: HOMO–LUMO gap [kcal/mol].

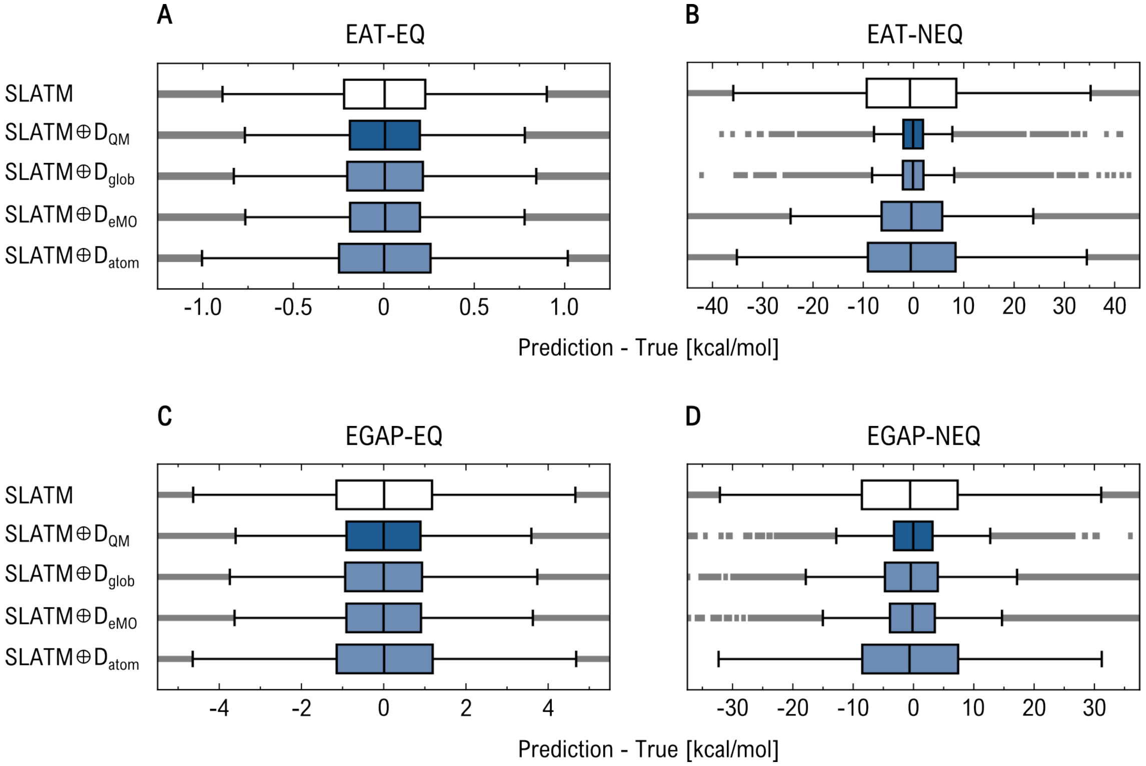

Fig. S2 Evaluation of atomization energy and HOMO–LUMO gap prediction models combining SLATM with subsets of the D_{QM} descriptor using Kernel Ridge Regression models trained on 16k samples. Panels **A** and **B** show residual distributions (prediction – true) for KRR atomization energy models on equilibrium and non-equilibrium geometries, respectively, using global (D_{glob}), molecular orbital energy (D_{eMO}), and atomic (D_{atom}) components. Panels **C** and **D** show the corresponding residual distributions for HOMO–LUMO gap.

5 Correlation between target properties and QM properties

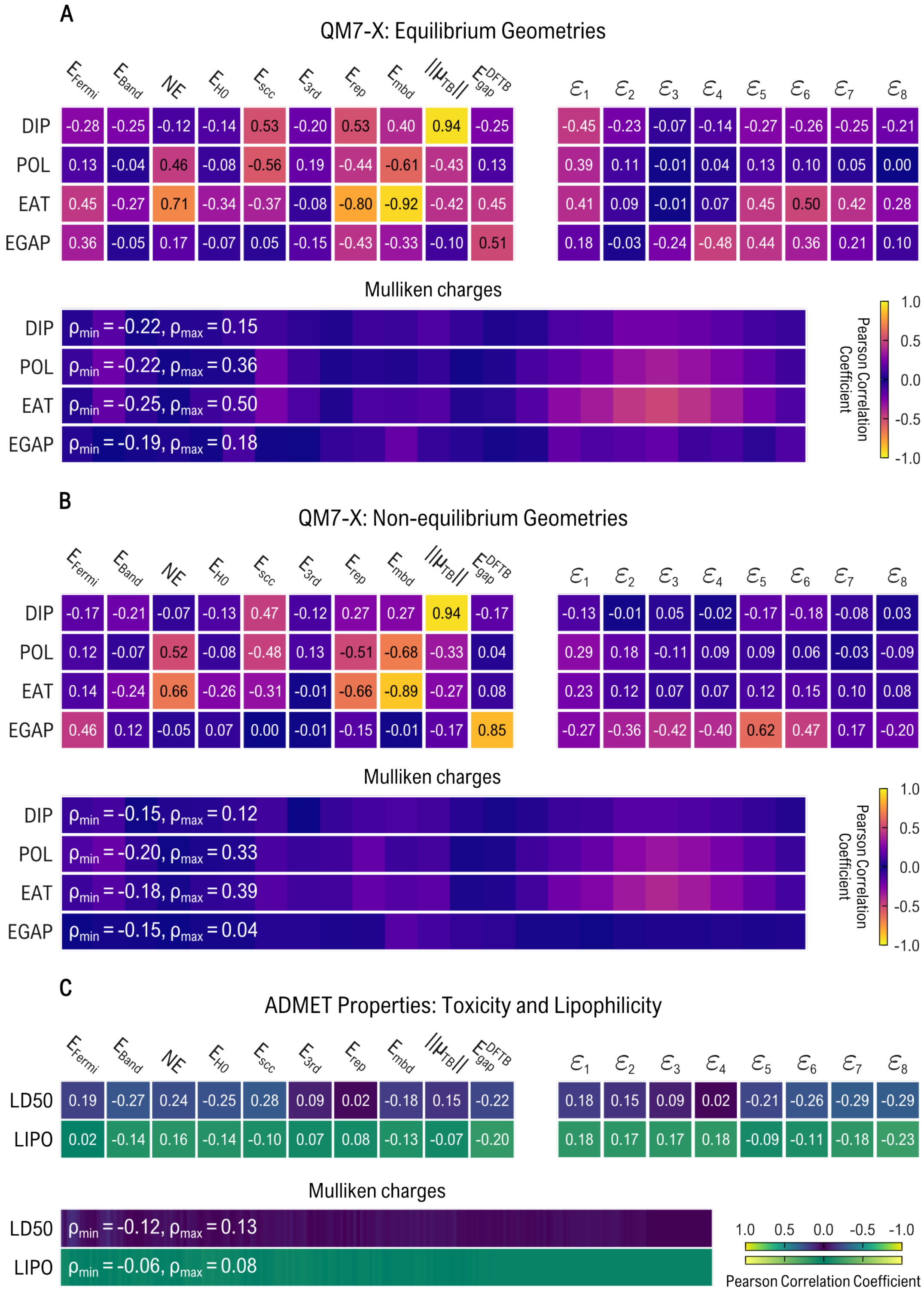

Fig. S3 Pearson correlation coefficients between the properties included in D_{QM} and the target properties: dipole moment (DIP), polarizability (POL), atomization energy (EAT), HOMO–LUMO gap (EGAP), toxicity (LD50), and lipophilicity (LIPO). For Mulliken charges, instead of reporting individual correlations, the minimum and maximum values across each set are provided.

6 Prediction of toxicity

Geom. Desc.	Metric	-	$\oplus D_{QM}$	$\oplus D_{glob}$	$\oplus D_{eMO}$	$\oplus D_{atom}$
BOB	MAE	0.476	**0.469**	0.479	0.480	0.476
	RMSE	0.643	0.635	0.629	0.631	**0.626**
	R2 score	0.544	**0.555**	0.534	0.532	0.539
SLATM	MAE	0.433	0.445	**0.426**	0.429	0.452
	RMSE	0.606	0.616	**0.597**	0.603	0.627
	R2 score	0.595	0.581	**0.607**	0.600	0.567

Table S7 Evaluation of toxicity prediction models using Kernel Ridge Regression, combining geometric descriptors with subsets of the D_{QM} descriptor: global (D_{glob}), molecular orbital energy (D_{eMO}), and atomic (D_{atom}) components.

7 Prediction of lipophilicity

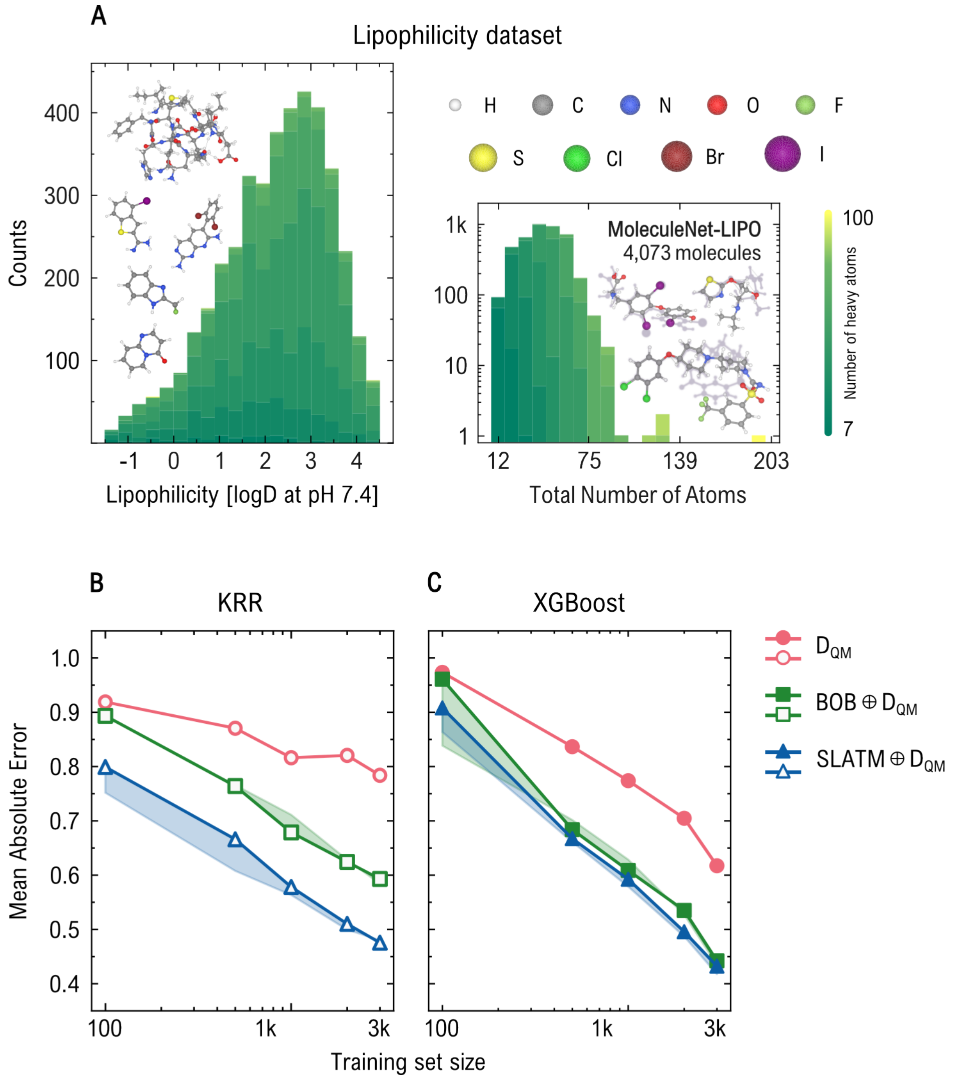

Fig. S4 Performance of models trained on the MoleculeNet–Lipophilicity dataset. Mean absolute errors (MAEs) obtained with D_{QM}, BOB $\oplus$ D_{QM}, and SLATM $\oplus$ D_{QM} are shown as solid lines, with shaded areas indicating improvements from adding D_{QM} to geometric descriptors. Panel **A** presents the property distribution, while panels **B** and **C** display the lipophilicity learning curves for Kernel Ridge Regression (KRR) and XGBoost models, respectively.

Geom. Desc.	Metric	-	$\oplus D_{QM}$	$\oplus D_{glob}$	$\oplus D_{eMO}$	$\oplus D_{atom}$
BOB	MAE	**0.584**	0.593	**0.584**	0.585	0.593
	RMSE	**0.751**	0.755	**0.751**	0.750	0.756
	R2 score	0.568	0.563	**0.569**	**0.569**	0.563
SLATM	MAE	0.480	**0.476**	0.480	0.478	0.479
	RMSE	0.679	**0.661**	0.679	0.673	**0.661**
	R2 score	0.624	0.643	0.624	0.630	**0.644**

Table S8 Evaluation of lipophilicity prediction models using Kernel Ridge Regression, combining geometric descriptors with subsets of the D_{QM} descriptor: global (D_{glob}), molecular orbital energy (D_{eMO}), and atomic (D_{atom}) components.

8 Computational costs within QUED framework

Figure S5 shows the computation times for each step of the QUED framework evaluated on 345 randomly selected molecules (165 from the toxicity dataset and 180 from the lipophilicity dataset). Model training times are excluded, as training costs depend strongly on hardware, hyperparameter optimization, and parallelization strategies.

The conformational search performed with CREST was run on a node equipped with 104 processors, and the computational cost is reported in CPU-hours in Panel **A**. This step represents the most computationally demanding component of the QUED workflow. We also calculated the computational time required to generate the electronic (D_{QM}) and geometric (BOB, SLATM, and SOAP) descriptors for the 345 randomly selected molecules. Because the configuration of the geometric representations (e.g., cutoff size and many-body terms) depends on the dataset, these timings are reported separately for each dataset, *i.e.*, Panel **B** for toxicity and Panel **C** for lipophilicity. The descriptor dimensionalities for the three datasets used in this work are reported in Table S9. Finally, Panels **D** and **E** present the time required to make a prediction using the best-performing models identified in this work. This inference time includes the generation of the molecular representation, loading of the trained model, and computation of the prediction.

As an illustrative example, we analyzed a subset of 137 molecules selected from the previously combined toxicity and lipophilicity datasets. These molecules contain between 30 and 60 atoms, and their SMILES representations are available in the QUED GitHub repository. For this subset, the conformational search required a total of 10,455 CPU-hours and generated 63,494 conformers. Of the 137 molecules, 76 were processed using the default CREST configuration described in the Methods section, including up to 10 metadynamics restart cycles. This group accounted for 8,465 CPU-hours. The remaining 61 molecules were treated using an accelerated setup, with reduced settings for iterative metadynamics genetic Z-matrix crossing (iMTD-GC) and molecular dynamics restricted to lower-energy conformers. These settings were enabled via the `mquick` and `norotmd` options in CREST, together with only five metadynamics restart cycles, resulting in a computational cost of 1,990 CPU-hours. The next step in QUED is the selection of the most stable conformers, those with the lowest DFTB3+MBD energies, and the corresponding calculation of descriptors. For this final set of 137 conformers, the generation of geometric descriptors requires a total of 6, 7, and 22 seconds for SOAP, BOB, and SLATM, respectively. In comparison, the computation of QM properties for the D_{QM} descriptor takes approximately 72 seconds. Thus, the cost of generating the electronic descriptor is comparable to that of SLATM and only about one order of magnitude higher than that of SOAP and BOB, indicating that it does not introduce a significant additional computational burden in the overall model construction.

Table S9 Dimensions of employed descriptors (i.e., number of entries in arrays).

	**Descriptor**			
**Dataset**	D_{QM}	BOB	SLATM	SOAP
QM7-X	40	528	17,895	8,272
Toxicity	213	39,349	79,945	22,680
Lipophilicity	220	24,753	58,786	18,396

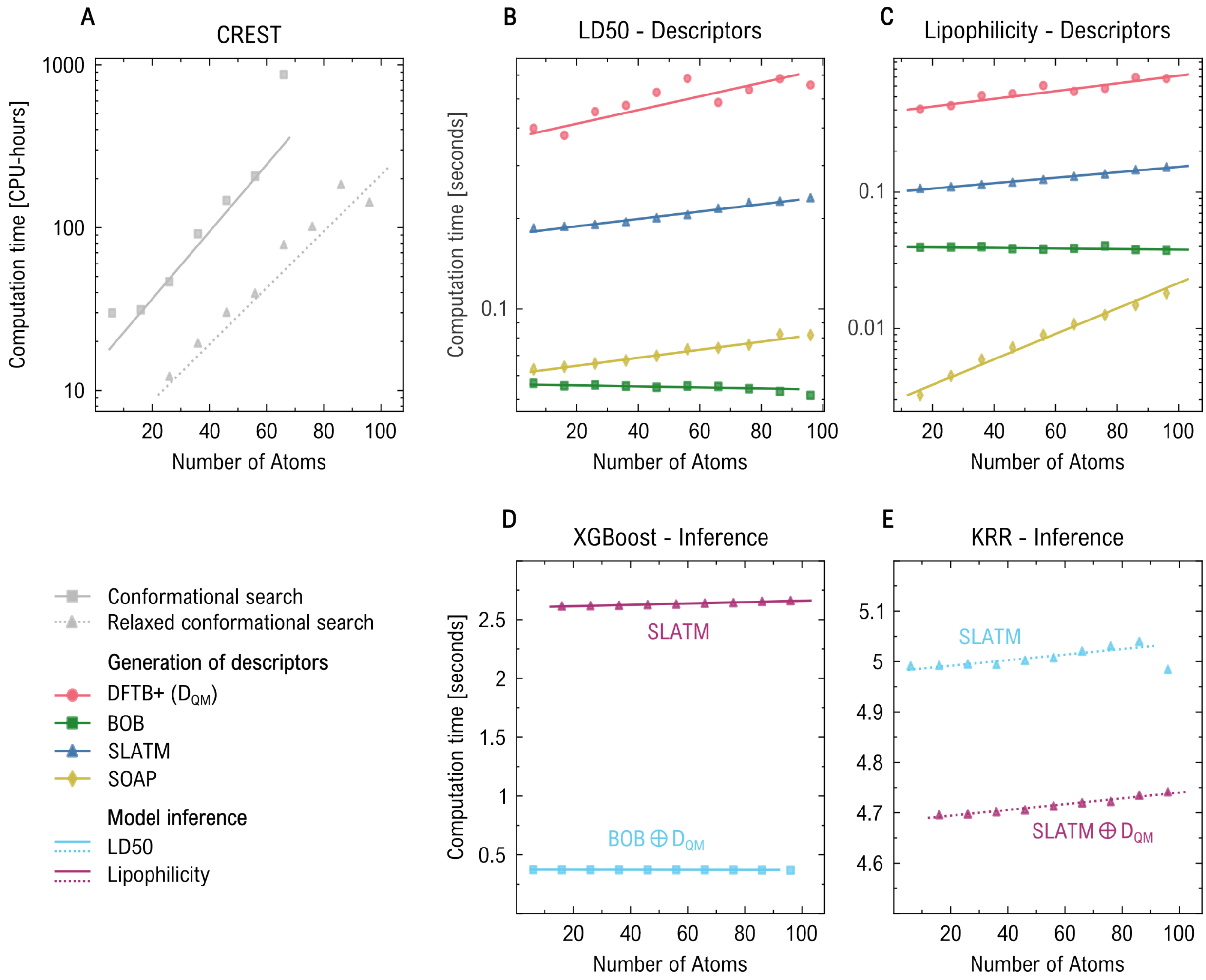

Fig. S5 Computation times for the different stages of the QUED framework as a function of molecular size, measured by the total number of atoms per molecule. Panel **A** shows the CPU hours required for the conformational search performed with the CREST code. Panels **B** and **C** report the time required to compute the electronic descriptor (D_{QM}, pink) and the geometric descriptors BOB (green), SLATM (blue), and Smooth Overlap of Atomic Positions (SOAP, yellow) for the toxicity and lipophilicity datasets, respectively. Panels **D** and **E** display the inference time of the best-performing ML models reported in this work.

9 Property prediction with SOAP and MACE

In addition to the models using the BOB and SLATM descriptors, we also tested the QUED framework with the Smooth Overlap of Atomic Positions (SOAP) descriptor. SOAP represents local atomic environments by calculating the overlap of Gaussian-smeared neighbor densities, producing a representation that is invariant to rotations, translations, and permutations, making it well-suited for ML models.

Table S10 summarizes the metrics obtained from XGBoost models trained with SOAP and SOAP⊕DQM. The SOAP descriptor was constructed using a cut-off radius of $rcut = 6.0$ Å, $n_{\max} = 8$ radial basis functions, and $l_{\max} = 6$ (the maximum degree of the spherical harmonics). This produces an atomic-level descriptor (i.e., a vector for each atom), which is then averaged over the power spectrum of different sites to yield a uniform-sized descriptor for each dataset.

Table S10 Mean absolute error (MAE), root-mean-squared error (RMSE), and R2 score values for direct learning of target properties via XGBoost with the SOAP descriptor. EAT: atomization energy [kcal/mol]; EGAP: HOMO–LUMO gap [kcal/mol]; POL: polarizability [a_0^3]; DIP: dipole moment [eÅ].

		MAE		RMSE		R2 score	
Target	Set	SOAP	SOAP⊕D_{QM}	SOAP	SOAP⊕D_{QM}	SOAP	SOAP⊕D_{QM}
EAT	EQ	4.926	**2.214**	9.194	**4.852**	0.998	**0.999**
	NEQ	14.586	**11.283**	18.837	**14.416**	0.989	**0.993**
EGAP	EQ	2.821	**1.442**	4.559	**2.207**	0.938	**0.985**
	NEQ	10.922	**4.071**	13.915	**5.480**	0.674	**0.949**
POL	EQ	0.406	**0.325**	0.693	**0.540**	0.995	**0.997**
	NEQ	1.156	**0.977**	1.537	**1.293**	0.981	**0.986**
DIP	EQ	0.046	**0.017**	0.073	**0.030**	0.924	**0.987**
	NEQ	0.129	**0.054**	0.173	**0.073**	0.646	**0.937**
Toxicity	Stable	**0.429**	0.436	**0.584**	0.601	**0.633**	0.625
Lipophilicity	Stable	0.465	0.465	0.611	**0.608**	0.728	0.728

Furthermore, we employed the state-of-the-art equivariant neural network architecture MACE to develop predictive models for our benchmark datasets. Our calculations focused solely on biological responses, since flexible molecules and intensive properties, such as lipophilicity and toxicity, present greater challenges for MACE. After tuning the cut-off radius in MACE models with 128 channels, third-degree correlations, two interaction blocks, and $l_{max} = 2$ (default parameters), the best-performing model achieved mean absolute errors (MAE) of 0.549 for toxicity and 0.458 for lipophilicity, using cut-off values of 6 Å and 4 Å, respectively (see Table S11). These errors are larger than those obtained with simpler geometric descriptors, such as BOB or SLATM, especially when combined with electronic descriptors.

Table S11 Mean absolute error (MAE) for direct learning of target properties using ML models trained with state-of-the-art equivariant neural network architecture MACE.

Target	cut-off	MAE
Toxicity	4.0	0.564
	5.0	0.553
	6.0	**0.549**
Lipophilicity	4.0	**0.458**
	5.0	0.474
	6.0	0.481
